# Supplementary material for: Separate brainstem circuits for fast steering and slow exploratory turns
Source: Nat Commun. 2025 Apr 3;16:3207. doi: 10.1038/s41467-025-58621-9 (PMC11968878; doi:10.1038/s41467-025-58621-9)
Supplement: Supplementary file 1 — Supplementary Information [file 41467_2025_58621_MOESM1_ESM.pdf]

Supplementary Figures and Figure Legends

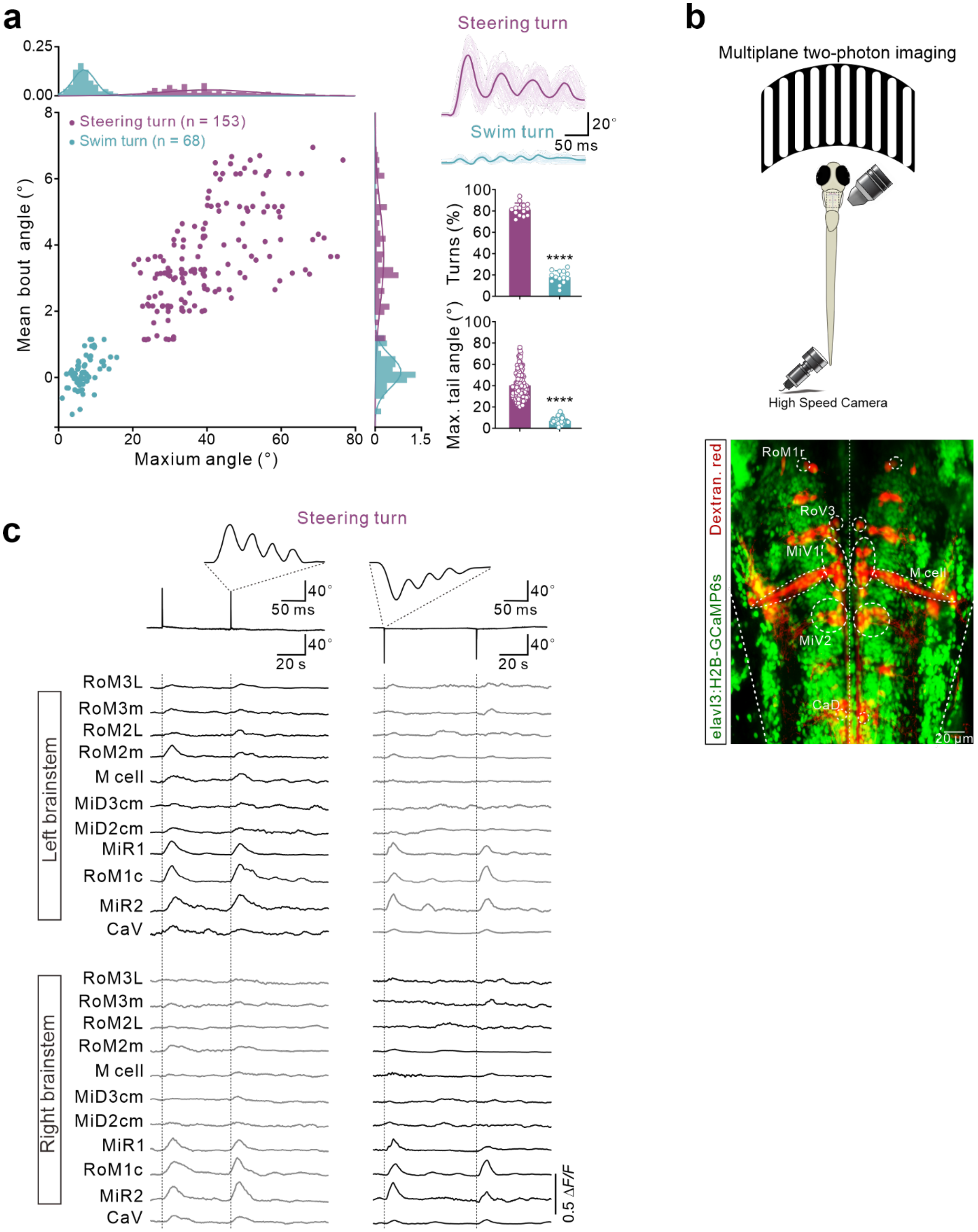

Supplementary Fig. 1 Brainstem SPNs that do not encode rapid steering turns.

(**a**) Turn movements induced in the dorsal-up preparations were classified by the classifier used in Fig. 1 into two distinct patterns: steering turns (plum, 153 events from 6 fish) and explorative turns (blue, 68 events from 6 fish). Each circle represents an individual event. Example traces of tail angle changes for both steering and explorative turns in the same preparation (top; 3 trials from 1 fish; 73 events classified as 48 steering turns and 25 explorative turns). Graph showing the occurrence of steering and explorative turns (middle; Unpaired *t*-test; 14 trials from 6 preparations) and tail angle (bottom) for both steering and explorative turns (Mann-Whitney test, 153 events for steering turns, 68 events for explorative turns; N = 6 fish). The color plum represents steering turns, while blue denotes explorative turns. All data are presented as mean ± SEM, ****$p < 0.0001$, ns denoting no significant difference. Each circle corresponds to an individual trial.

(**b**) Experimental configuration for two-photon calcium imaging of brainstem neurons in an in vivo ventral-up preparation, with an optic flow grid positioned in front of the preparation. A high-speed camera is used for simultaneous video tracking of turning movements (upper panel). The spinal-projecting neurons (SPNs) in specific brainstem nuclei are identified through retrograde labeling using rhodamine dextran in the Tg(elavl3: H2B-GCaMP6s) fish line (lower panel).

(**c**) Two-photon calcium imaging experiments (Fig. 2) showing the calcium response of the indicated SPNs during a steering turn. The other SPNs did not show a significant response to the steering turns.

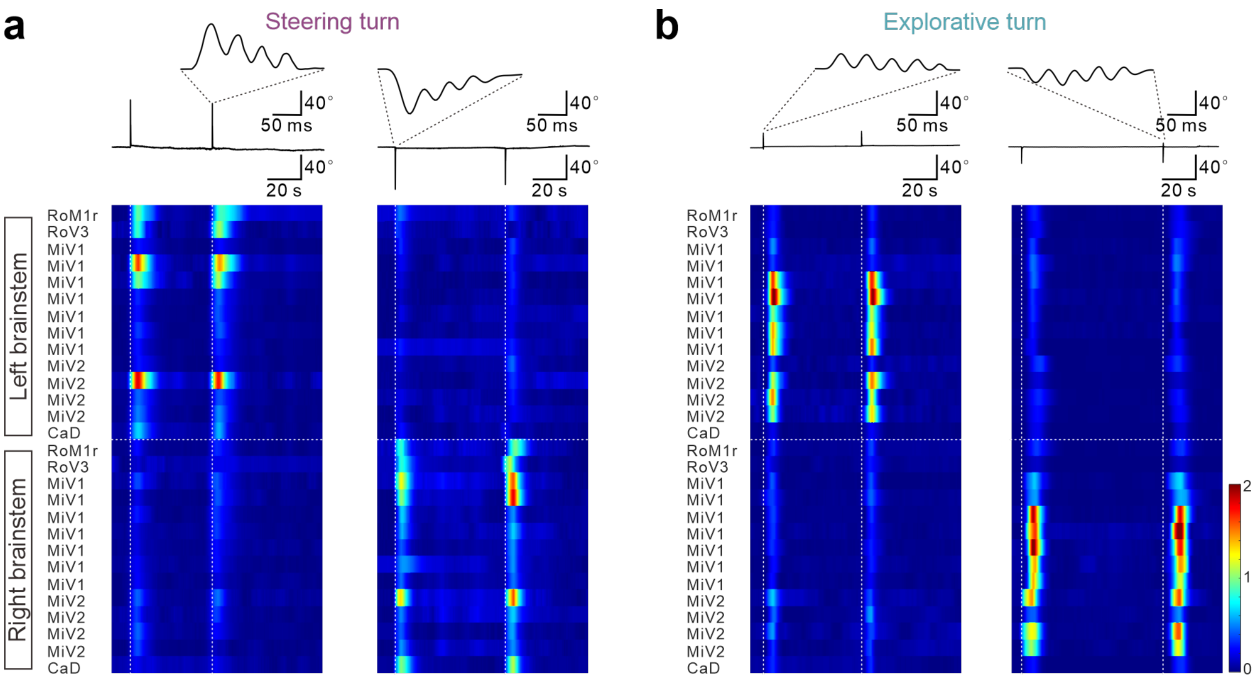

Supplementary Fig. 2 The activity of brainstem neurons during steering turn and exploratory turn.

(**a**) Heatmap graph showing the spinal projecting neurons (SPNs) in distinct nuclei of the brainstem recruited during rapid steering turns. Excitatory neurons (2 neurons in the rostral part of MiV1; 1 neuron in the rostral part of MiV2; 1 neuron in RoM1r, and 1 neuron in RoV3) and an inhibitory neuron (1 neuron in CaD) displayed significantly larger calcium responses during ipsilateral rapid steering turns, while the rest of the neurons showed weak or no response. This graph refers to Fig. 2b.

(b) Heatmap graph showing the spinal projecting neurons (SPNs) in the brainstem recruited during explorative turns. Excitatory neurons in the caudal part of both MiV1 and MiV2 displayed significantly larger calcium responses during ipsilateral explorative turns, while the rest of the neurons showed weak or no response. This graph refers to Fig. 2e.

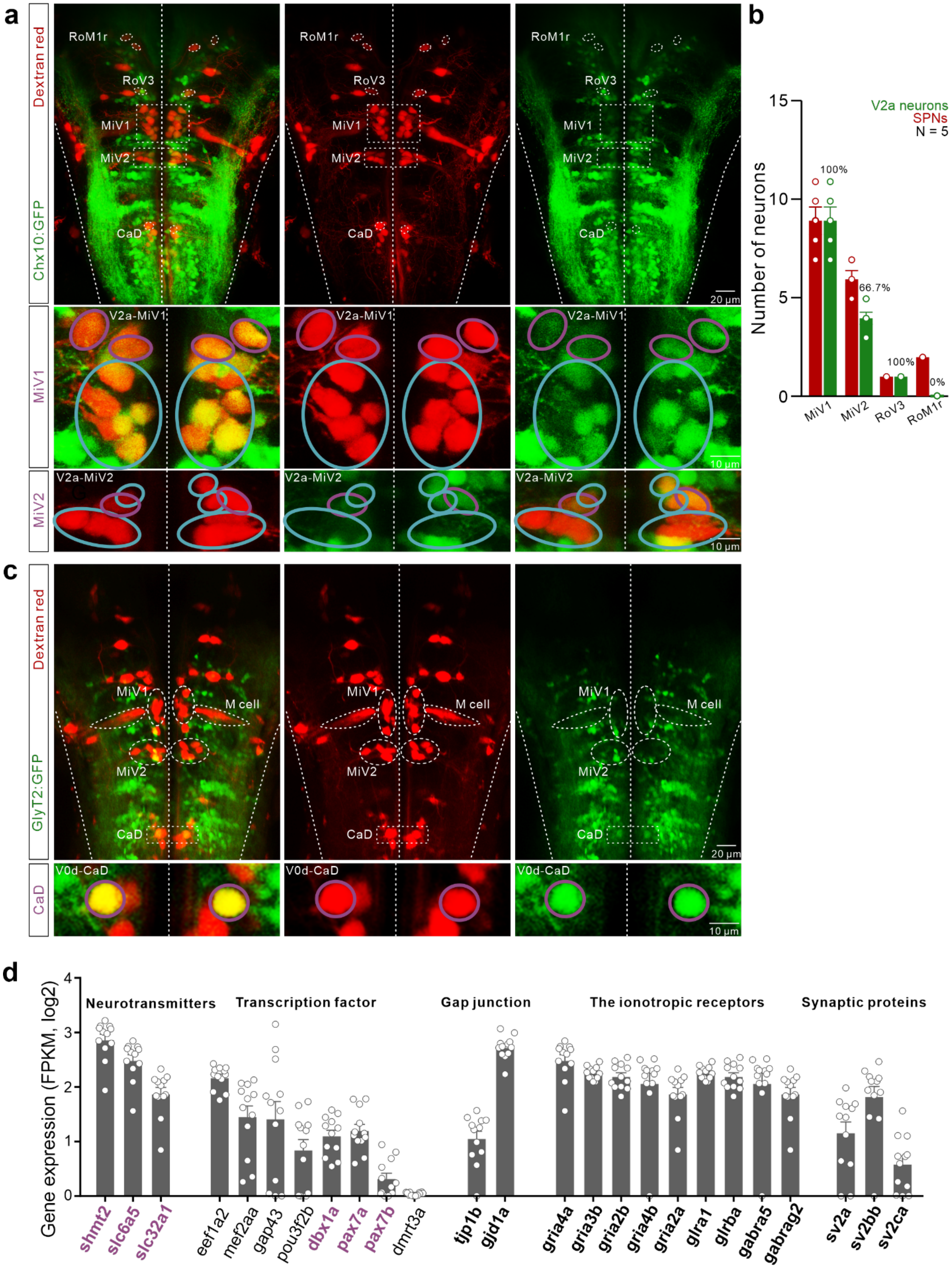
a
Dextran red
Chx10:GFP
RoM1r
RoV3
MiV1
MiV2
CaD
20 μm
V2a-MiV1
10 μm
V2a-MiV2
b
V2a neurons
SPNs
N = 5
Number of neurons
15
10
5
0
100%
66.7%
100%
0%
MiV1
MiV2
RoV3
RoM1r
c
GlyT2:GFP
M cell
V0d-CaD
d
Gene expression (FPKM, log2)
4
3
2
1
0
Neurotransmitters
Transcription factor
Gap junction
The ionotropic receptors
Synaptic proteins
shmt2
slc6a5
slc32a1
eef1a2
mef2aa
gap43
pou3f2b
dbx1a
pax7a
pax7b
dmrt3a
tjp1b
gjd1a
gria4a
gria3b
gria2b
gria4b
gria2a
glra1
glrba
gabra5
gabrag2
sv2a
sv2bb
sv2ca

Supplementary Fig. 3 Molecular identity of the SPNs encoding turning movements.

(**a**) The stacked image (upper panel) identifies the spinal-projecting neurons (SPNs) in the RoV3, MiV1, and MiV2 nuclei that encode turning movements as Chx10-expressing V2a neurons. The enlargement of the dashed-line rectangular box displays the SPNs retrogradely labeled with dextran red in the MiV1 and MiV2 nuclei, which are V2a neurons (lower panel).

(**b**) Statistical analysis showing the percentage of V2a neurons in the different brainstem nuclei: 100% in MiV1, 66.7% in MiV2, 100% in RoV3, and 0% in RoM1r (all bars represent mean ± SEM, $N = 5$ fish).

(**c**) The stacked image identifies the glycinergic neurons in the CaD nucleus, labeled with dextran red in the Tg(*GlyT2:GFP*) line (upper panel). An enlargement of the dashed box in **b** (lower panel).

(**d**) The transcriptomic profile of steering V0d neurons in the CaD nucleus, as revealed by single-neuron Smart-seq2. The graph displays quantitative analysis for neurotransmitter genes, transcription factor genes, gap junction genes, ionotropic receptor genes, and synaptic protein genes (data is represented as mean ± SEM; $n = 12$ neurons from 12 preparations).

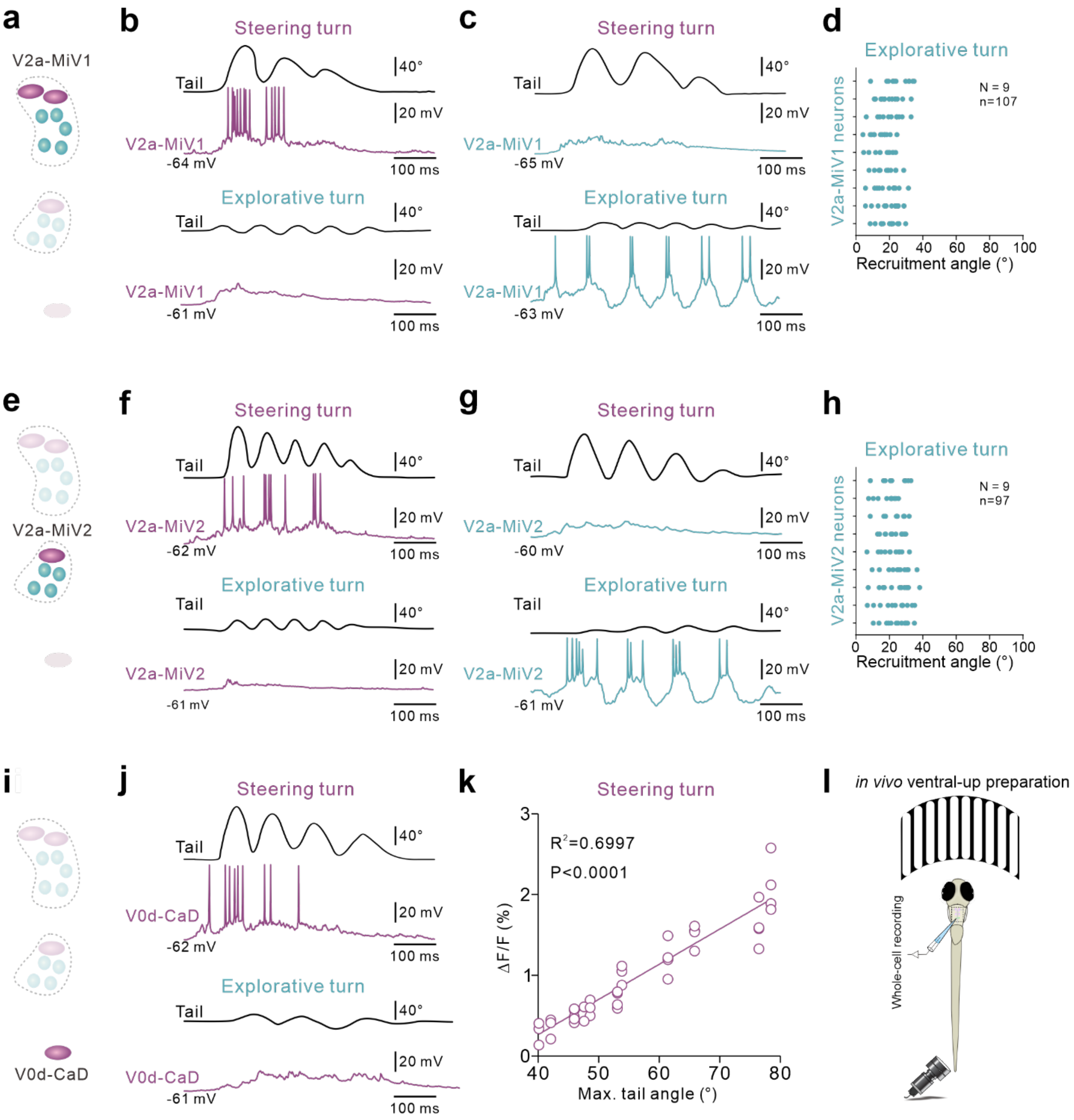

Supplementary Fig. 4 Distinct brainstem circuits for rapid steering and slow exploratory turns.

(**a-d**) Whole-cell patch-clamp recordings of V2a neurons in the MiV1 nucleus (**a**). Steering V2a neurons are activated during rapid steering turns but not during exploratory turns (**b**, **c**). Exploratory V2a neurons in the MiV1 nucleus are activated during slow exploratory turns but not during steering turns (**b**, **c**). The graph shows that exploratory V2a neurons are recruited at low-

amplitude tail bending angles, typical of exploratory turns (**d**; *N* = 9 neurons from 9 preparations, *n* = 107 events). All graphs display mean ± SEM; circles represent individual events.

(**e**-**h**) Whole-cell patch-clamp recordings of V2a neurons in the MiV2 nucleus (**e**). Steering V2a neurons are recruited during steering turns but not during exploratory turns (**f**, **g**). Exploratory V2a neurons in the MiV2 nucleus are recruited during exploratory turns but not during steering turns (**f**, **g**). The graph shows that exploratory V2a neurons are recruited at low-amplitude tail bending angles during exploratory turns (**h**; *N* = 9 neurons from 9 preparations, *n* = 97 events). All graphs show mean ± SEM; circles represent individual events.

(**i**-**j**) Whole-cell patch-clamp recordings of V0d neurons in the CaD nucleus (**i**). Steering V0d neurons are recruited during steering turns, but not during exploratory turns (**i**, **j**).

(**k**) A linear correlation exists between the calcium response in the steering V2a/V0d neurons and maximum tail bending angle.

(**l**) Schematic illustration depicts the in vivo ventral-up preparation, showing the optic flow grid positioned in front of the zebrafish and a high-speed camera used to monitor tail movements. Whole-cell patch-clamp recordings were performed in this preparation.

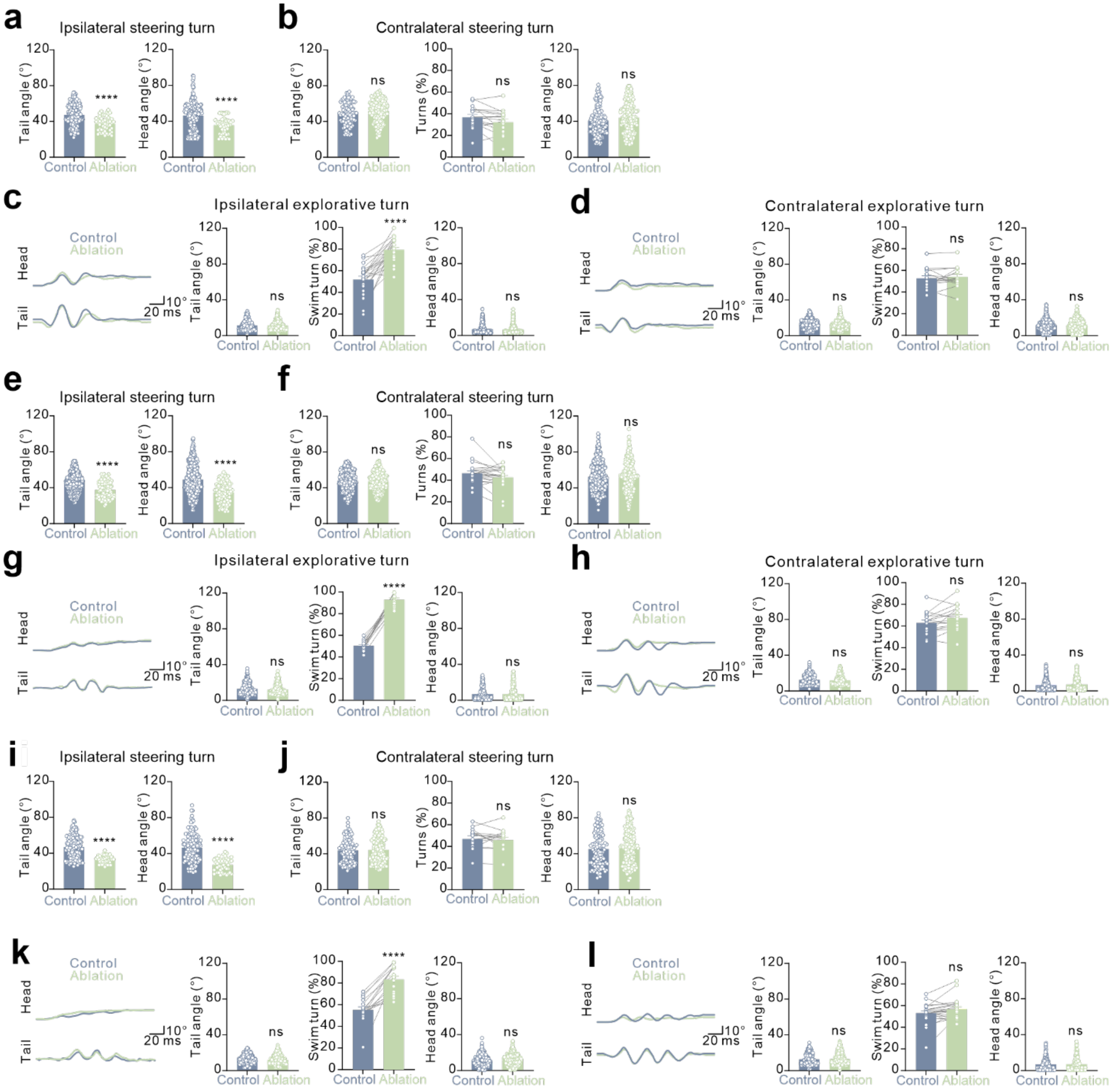

Supplementary Fig. 5 Effect of ablation of steering circuit neurons.

(**a**-**d**) Ablation of steering V2a neurons in the MiV1 and MiV2 nuclei increased the occurrence of ipsilateral exploratory turns, but did not affect the locomotor parameters of either steering turns or exploratory turns (*N* = 16 fish).

(**e**-**h**) Ablation of steering V0d neurons in the CaD nucleus increased the occurrence of ipsilateral exploratory turns but, did not affect the locomotor parameters of either steering turns or exploratory turns (*N* = 22 fish).

(**i**-**l**) Ablation of both steering V2a neurons in the MiV1 and MiV2 nuclei, as well as steering V0d neurons in the CaD nucleus, increased the occurrence of ipsilateral exploratory turns, but did not affect the locomotor parameters of either steering turns or exploratory turns (*N* = 16 fish). All graphs display mean ± SEM, Student's *t*-test, ****$p < 0.0001$, and ns denoting no significant difference. Each circle represents an individual fish.

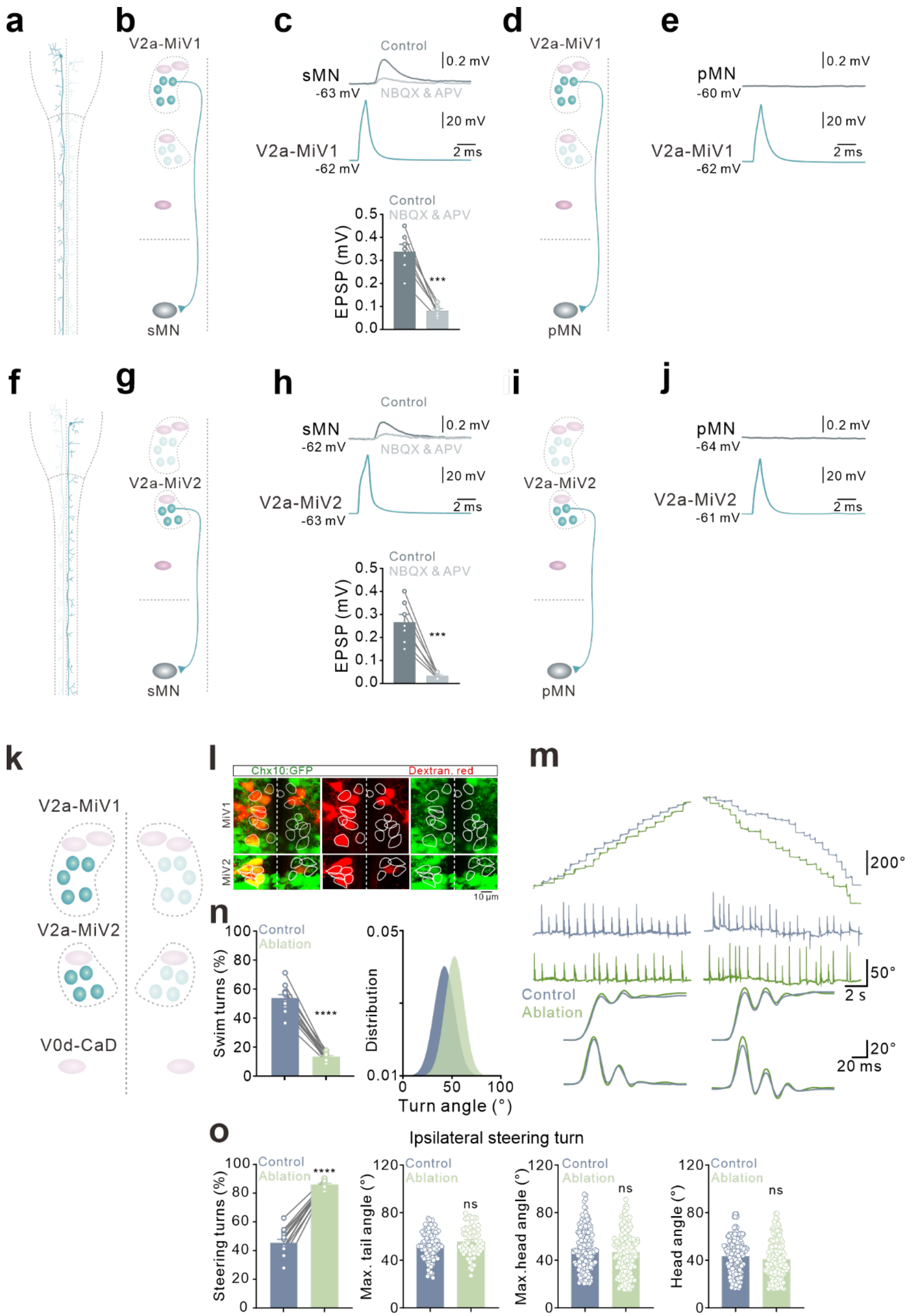

a
b
V2a-MiV1
sMN
c
Control
sMN
-63 mV
NBQX & APV
0.2 mV
20 mV
V2a-MiV1
-62 mV
2 ms
EPSP (mV)
***
d
V2a-MiV1
pMN
e
pMN
-60 mV
0.2 mV
20 mV
V2a-MiV1
-62 mV
2 ms
f
g
V2a-MiV2
sMN
h
Control
sMN
-62 mV
NBQX & APV
V2a-MiV2
-63 mV
i
V2a-MiV2
pMN
j
pMN
-64 mV
V2a-MiV2
-61 mV
k
V2a-MiV1
V2a-MiV2
V0d-CaD
l
Chx10:GFP
Dextran. red
MiV1
MiV2
10 µm
m
200°
50°
2 s
Control
Ablation
20°
20 ms
n
Swim turns (%)
****
Distribution
Turn angle (°)
o
Ipsilateral steering turn
Steering turns (%)
Max. tail angle (°)
Max.head angle (°)
Head angle (°)
ns

Supplementary Fig. 6 Morphology and loss-of-function of V2a neurons encoding explorative turns.

(a-e) Morphology of a V2a neuron in the MiV1 nucleus, which encodes explorative turns (**a**). Activation of a V2a neuron elicited monosynaptic EPSPs in secondary motor neurons, which were blocked by NBQX and AP5 (**b, c**; grey trace). Graph presents the statistical analysis (**c**; Paired *t*-test, *n* = 7 pairs from 7 preparations). V2a neuron in the MiV1 nucleus did not elicit monosynaptic EPSPs in a primary motor neuron (**d, e**; *N* = 7 pairs from 7 preparations).

(f-j) Morphology of a V2a neuron in the MiV2 nucleus that is active during explorative turns (**f**). Activation of a V2a neuron produced monosynaptic EPSPs in secondary motor neurons, which were blocked by NBQX and AP5 (**g, h**; grey trace). Graph presents the statistical analysis (**h**; Paired *t*-test, *n* = 7 pairs from 7 preparations). Lack of monosynaptic connections between a V2a neuron in the MiV2 nucleus active during explorative turns and a primary motor neuron (**i, j**; *N* = 7 pairs from 7 preparations).

(k-o) Unilateral ablation of V2a neurons active during explorative turns in the MiV1 and MiV2 nuclei (**k**). Image stacks confirm the unilateral ablation of these V2a neurons through retrograde tracing in the Tg(*Chx10:GFP*) fish line (**l**). The unilateral ablation of these V2a neurons significantly reduced the cumulative angle of heading directional changes, as well as the tail angle during ipsilateral explorative turns (**m**). Statistical analysis shows that, compared to the control group, there was a significantly decreased in the occurrence of steering turns and the distribution of tail angle amplitude following the ablation of the steering V2a neurons (**n**) (Paired *t*-test, *N* = 12 fish). Statistical analysis shows that, in comparison to the control group, the ablation of V2a neurons in the MiV1 and MiV2 nuclei increased the occurrence of steering turns, but it did not affect the associated locomotor parameters (**o**) (Paired *t*-test, *N* = 12 fish).

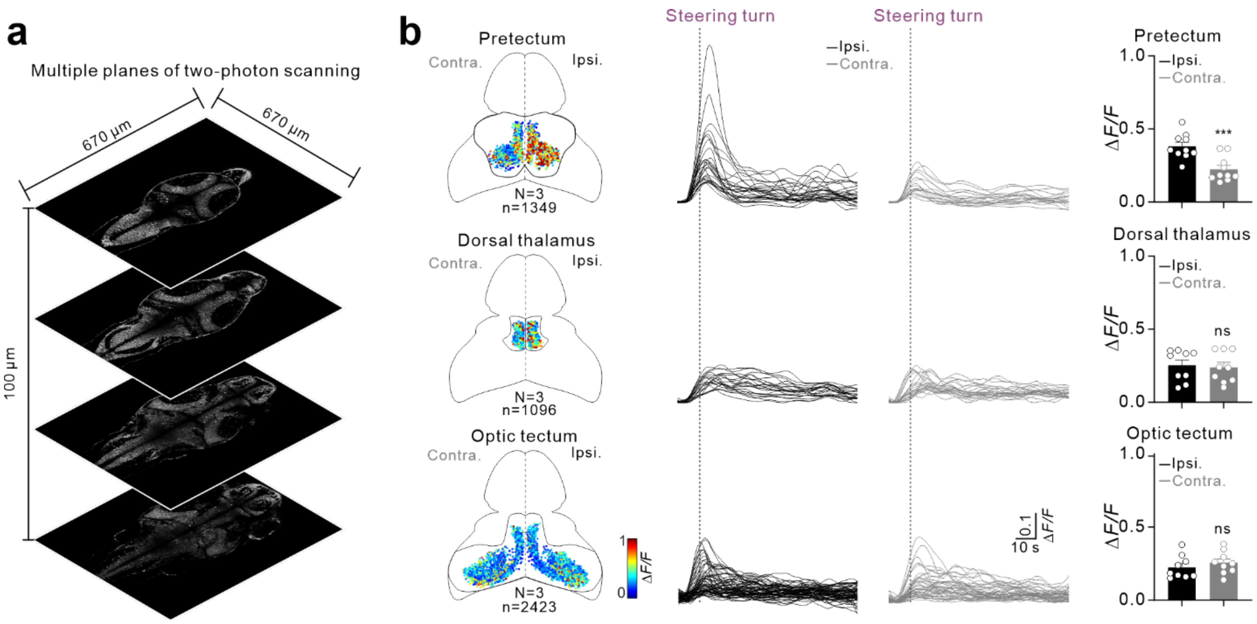

Supplementary Fig. 7 Neurons in the pretectum exhibit direction selectivity during steering turns, in contrast to those in the dorsal thalamus and optic tectum.

(**a**) Multiplane two-photon scanning in the pretectum, thalamus, and optic tectum.

(**b**) During asymmetrical optic flow, neurons in the pretectum exhibited significant direction selectivity (upper panel), while neurons on both sides of the dorsal thalamus and optic tectum showed uniform calcium responses during steering turns (middle and lower panels). All graphs display mean ± SEM, Student's *t*-test, ****p* < 0.001, and ns denotes no significant difference, with each circle representing an individual trial.

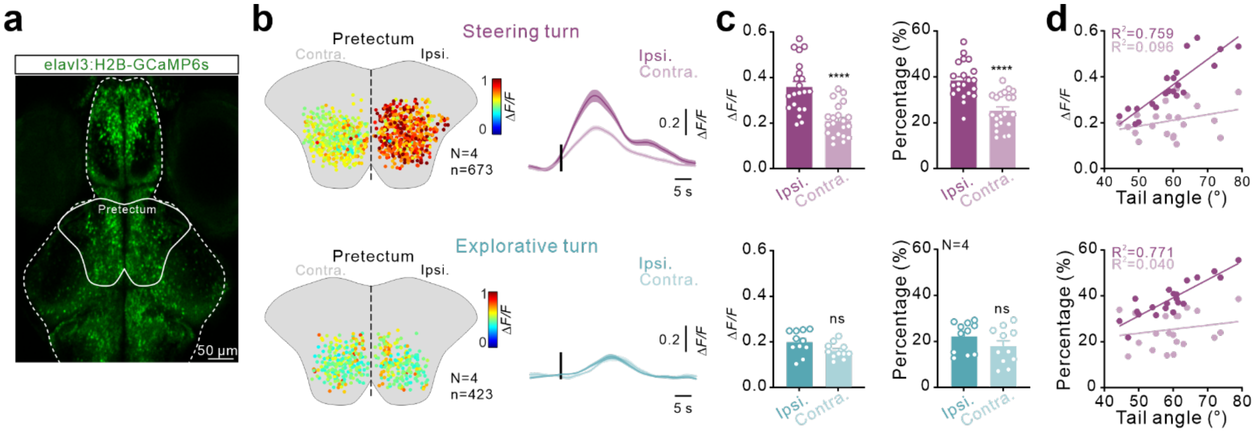

Supplementary Fig. 8 Asymmetric activation of neurons in the ventral pretectum during steering turns.

(**a**) Image showing pretectal neurons in the Tg (*elavl3:H2B-GCaMP6s*) fish line.

(**b**-**c**) Statistical analysis of soma distribution and response strength of recruited pretectal neurons during steering turns (upper left panel in **b**) and during exploratory turns (lower left panel in **b**). Average response in ipsilateral pretectum neurons was significantly larger than those in the contralateral pretectum during steering turns (upper right panel in **b**). There was no significant difference between the responses of pretectal V2a neurons in the ipsilateral and contralateral pretectum during exploratory turns (lower right panel in **b**). Population data for **b** (**c**; unpaired *t*-test, *N* = 6 fish, with 21 trials for steering turns and 11 trials for exploratory turns).

(**d**) The increase in calcium response ($\Delta F/F$) and the percentage of ipsilateral recruited pretectal neurons, but not the contralateral ones, displayed a linear relationship with the increase in the amplitude of the tail bending angle during steering turns (*N* = 6 fish, 21 trials from 6 fish for steering turns and 11 trials from 6 fish for exploratory turns; each circle represents an individual trial).

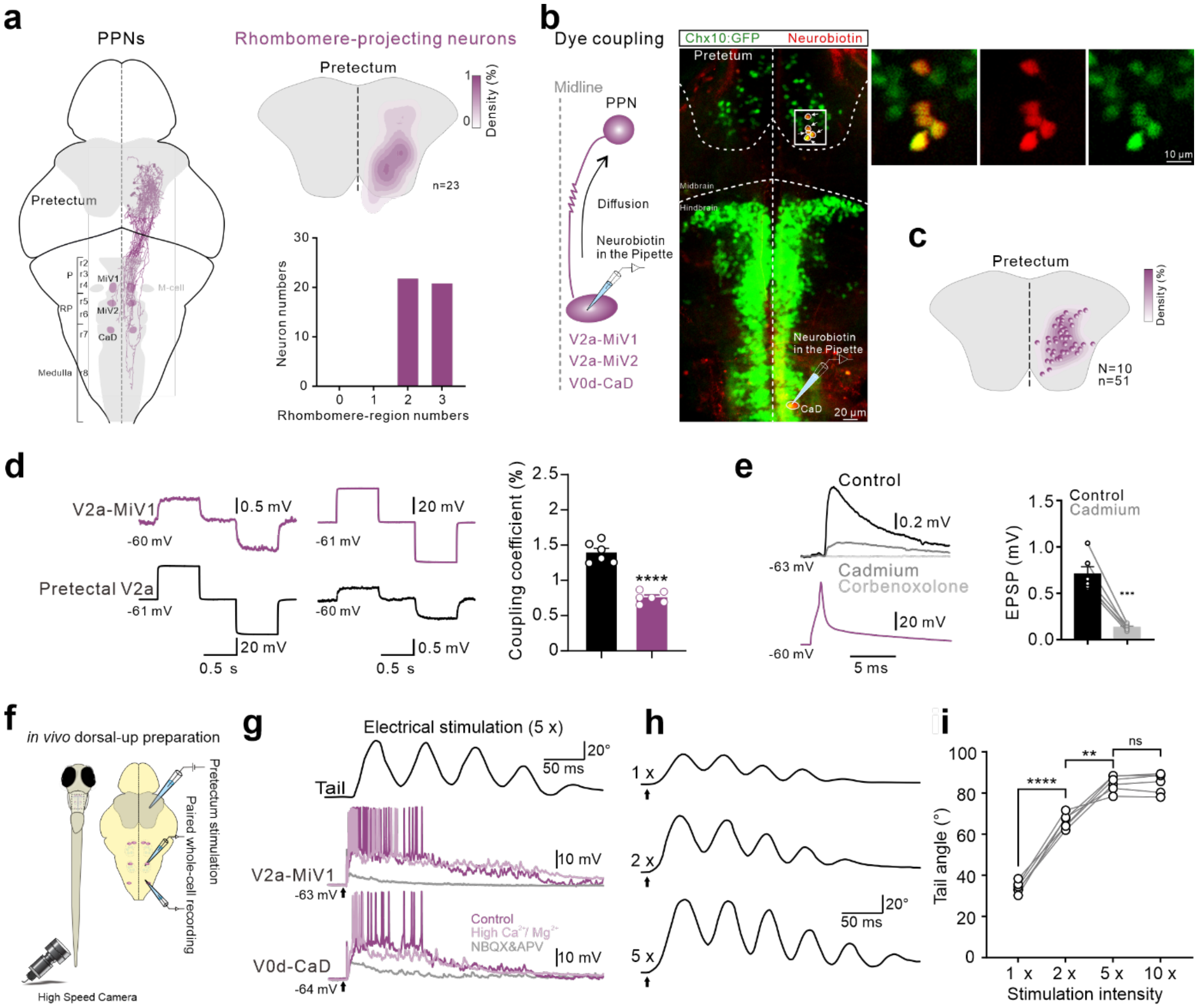

Supplementary Fig. 9 Neurons in the ventral pretectum drive brainstem steering circuit and generate steering turns.

(**a**) Axons of pretectal projecting neurons (PPNs) extended their projections across the MiV1, MiV2, and CaD nuclei (https://mapzebrain.org/atlas/3d). The spatial distribution of the PPN somata were located in the ventral pretectum.

(**b**-**c**) Neurobiotin in the recorded steering V2a or V0d neurons diffused to the pretectal V2a neurons via dye coupling in the Tg (*Chx10:GFP*) fish line. The image within the box was enlarged (upper right panel). Spatial distribution of dye-coupled PPNs located in the ventral pretectum (**c**).

(**d**-**e**) Electrical coupling between pretectal V2a neurons and steering V2a neurons in the MiV1 nucleus (**d**; Unpaired *t*-test, *n* = 6 pairs from 6 preparations). Pretectal V2a neurons induced monosynaptic EPSPs in the steering V2a neurons. The chemical component of the EPSPs was inhibited by cadmium and the remaining electrical component was completely blocked by arbenoxolone (**e**; paired *t*-test, *n* = 6 pairs from 6 preparations). All graphs display mean ± SEM, ****p < 0.0001, ***p < 0.001, ns denoting no significant difference.

(**f**-**i**) Experimental setup (**f**). Electric stimulation of the ventral pretectum simultaneously activated the steering V2a and V0d neurons, resulting in a steering turn. The discharge of steering neurons induced by electrical stimulation persisted even after the application of a high Ca^{2+}/Mg^{2+} solution but was blocked by the addition of NBQX and AP5 (**g**). The amplitude of the tail angle increased with stimulation intensity and eventually reached a plateau (**h**-**i**; one-way ANOVA, *N* = 6 fish, each circle corresponds to an individual preparation). All graphs display mean ± SEM, ***p < 0.001, **p < 0.01, ns denotes no significant difference.

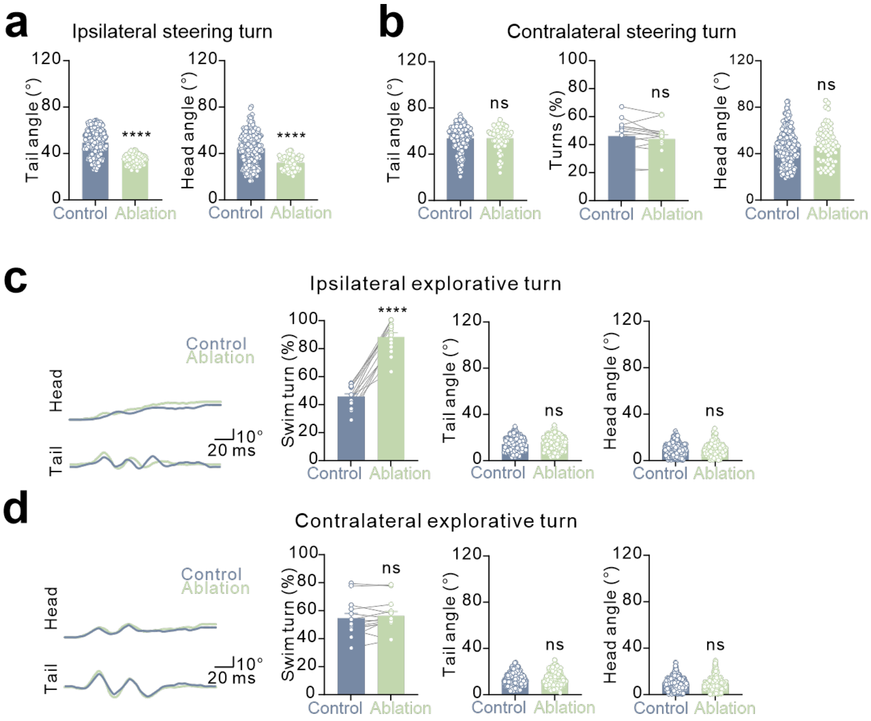

Supplementary Fig. 10 Ablation of the pretectal V2a neurons affected the execution of steering turns.

(**a**-**d**) In freely moving zebrafish, unilateral ablation of pretectal V2a neurons reduced both the tail angle and head angle during ipsilateral steering turns (**a**) but did not affect the locomotor parameters of the contralateral steering turns (**b**) or exploratory turns (**c**, **d**). Laser ablation of pretectal V2a neurons increased the occurrence of ipsilateral exploratory turns, while contralateral exploratory turns remained unchanged (**c**, **d**).
